# A Thermostable *Aspergillus fumigatus* GH7 Endoglucanase Over-Expressed in *Pichia pastoris* Stimulates Lignocellulosic Biomass Hydrolysis

**DOI:** 10.3390/ijms20092261

**Published:** 2019-05-07

**Authors:** Aline Vianna Bernardi, Deborah Kimie Yonamine, Sergio Akira Uyemura, Taisa Magnani Dinamarco

**Affiliations:** 1 Faculty of Philosophy, Sciences and Literature of Ribeirão Preto, Chemistry Department, University of São Paulo, Ribeirão Preto 14040-901, Brazil; alinevbernardi@gmail.com (A.V.B.); deborah.yonamine@hotmail.com (D.K.Y.); 2 Faculty of Pharmaceutical Science, Department of Clinical, Toxicological and Bromatological Analysis, University of São Paulo, Ribeirão Preto 14040-903, Brazil; suyemura@fcfrp.usp.br

**Keywords:** Af-EGL7, GH7 endoglucanase, biomass hydrolysis, thermostability

## Abstract

In the context of avoiding the use of non-renewable energy sources, employing lignocellulosic biomass for ethanol production remains a challenge. Cellulases play an important role in this scenario: they are some of the most important industrial enzymes that can hydrolyze lignocellulose. This study aims to improve on the characterization of a thermostable *Aspergillus fumigatus* endo-1,4-β-glucanase GH7 (Af-EGL7). To this end, Af-EGL7 was successfully expressed in *Pichia pastoris* X-33. The kinetic parameters *K_m_* and *V_max_* were estimated and suggested a robust enzyme. The recombinant protein was highly stable within an extreme pH range (3.0–8.0) and was highly thermostable at 55 °C for 72 h. Low Cu^2+^ concentrations (0.1–1.0 mM) stimulated Af-EGL7 activity up to 117%. Af-EGL7 was tolerant to inhibition by products, such as glucose and cellobiose. Glucose at 50 mM did not inhibit Af-EGL7 activity, whereas 50 mM cellobiose inhibited Af-EGL7 activity by just 35%. Additionally, the Celluclast^®^ 1.5L cocktail supplemented with Af-EGL7 provided improved hydrolysis of sugarcane bagasse “in natura”, sugarcane exploded bagasse (SEB), corncob, rice straw, and bean straw. In conclusion, the novel characterization of Af-EGL7 conducted in this study highlights the extraordinary properties that make Af-EGL7 a promising candidate for industrial applications.

## 1. Introduction

Raw material processing generates tons of agricultural and industrial waste every year. Unfortunately, biomass accumulation and misuse of such materials cause serious environmental issues. In addition, increased energy consumption, depletion of fossil fuel sources, and the need to abate global warming have drawn special attention to a new generation of renewable energy [1,2,3]. In this scenario, reusing lignocellulosic residues to synthesize chemical compounds and high-value products like biofuels and other green chemicals has emerged as one of the most potential strategies to overcome environmental problems [2,4].

Lignocellulose consists mainly of cellulose (35–50%), hemicellulose (20–30%), and lignin (20–30%) [5]. Cellulases produced by fungi and bacteria can degrade cellulose [6,7]. In fact, cellulases are considered to be some of the most important industrial enzymes as they can convert cellulose to sugars that can be further fermented to generate bioethanol and biobased products [6,8,9]. The classic mechanism of substrate degradation by cellulases involves the synergistic action of three types of hydrolytic enzymes: endo-1,4-β-glucanases (EC 3.2.1.4), cellobiohydrolases/exoglucanases (EC 3.2.1.91 and EC 3.2.1.176), and β-glucosidades (EC 3.2.1.21) [8,9].

Endo-1,4-β-glucanases catalyze the initial attack on the cellulose fibrils and randomly cleave the β-1,4-glycosidic bonds present in the amorphous regions of the cellulose chain. The other two cellulases then act to convert the oligosaccharides released from this hydrolysis into glucose [10,11]. According to the carbohydrate-active enzymes (CAZymes) database, endo-1,4-β-glucanases are widespread and are classified into 16 glycosyl hydrolase (GH) families. Enzymes belonging to the GH7 family can act on a broad range of substrates, including cellulose, β-glucan, lichenin, laminarin, and even xylan. This lack of specificity makes the endo-1,4-β-glucanases of this family very attractive for various industrial applications [9].

Because enzymes need to be exposed to extreme conditions during several industrial processes, stability to high temperatures within a wide pH range, as well as tolerance to inhibition by reaction products, are the most desired enzymatic properties at the industrial level [9,12]. Biomass saccharification at elevated temperatures reduces polysaccharides viscosity and bacterial contamination risks and allows enzymes to be directly used after steam pre-treatment, thereby shortening process duration and saving energy [11,13].

*Aspergillus fumigatus*, a thermophilic fungus, secretes several CAZymes that can be further used in enzymatic cocktails. Therefore, this fungus plays an important role in biomass deconstruction [4,7,14]. In our previous work, we expressed recombinant Af-EGL7 in *E. coli* and characterized its function [4]. Compared to the prokaryotic system, heterologous expression in the methylotrophic yeast *P. pastoris* offers many advantages, especially in terms of recombinant protein processing, folding, and post-translational modifications, which can influence enzyme functioning and even stability [15,16].

This paper reports on the successful gene *Af-egl7* cloning and expression in *P. pastoris* X-33 and on the characterization of the purified Af-EGL7. It shows that a recombinant GH7 endo-1,4-β-glucanase, with higher pH and thermal stabilities, is more active on complex biomasses. Our results highlight the industrial potential of the *A. fumigatus* recombinant endoglucanase Af-EGL7.

## 2. Results and Discussion

### 2.1. Af-EGL7 Cloning and Expression

Here, the CPEC method was used to amplify the endo-1,4-β-glucanase ORF (Open reading frame) *Af-egl7* (Afu6g01800) from the *A. fumigatus* cDNA and to clone it into the expression vector pPICZαA [17]. The recombinant vector pPICZαA/*Af-egl7* was transformed into *E. coli* DH10β for plasmid propagation. Sequencing was performed to analyze possible mutations in the *Af-egl7* sequence and to verify if the sequence had been cloned in frame with α factor signal peptide, which is important to obtain the recombinant enzyme extracellularly in the culture broth.

**Figure 1:**
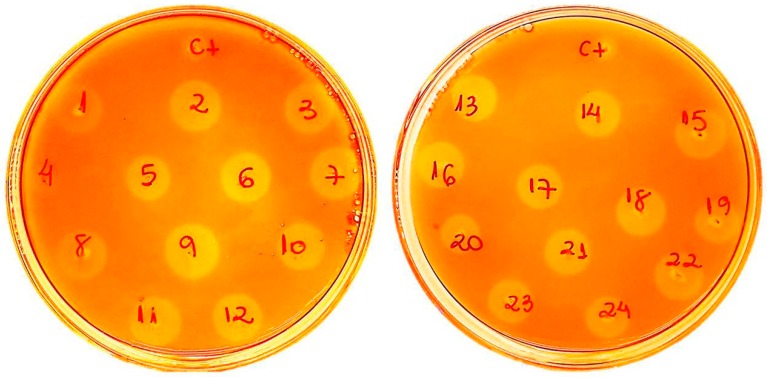
Screening of Af-EGL7 activity in 24 *Pichia pastoris* transformants on 1% (*w*/*v*) CM-Cellulose plate after heterologous expression induction with 1% (*v*/*v*) methanol. The host transformed with empty vector was used as control (ct). Pale orange halos bigger than the control indicated the positive functional colonies.

Next, the recombinant plasmid pPICZαA/*Af-egl7* was linearized with the restriction enzyme *Pme*I to enable its integration into the *P. pastoris* X-33 genome by homologous recombination, and transformed into this strain. After growth at 30 °C for three days, positive transformants were selected with the Zeocin resistance marker. The 24 selected positive transformants were screened in solid medium containing 1% (*w*/*v*) CM-Cellulose as a substrate, and 1% (*v*/*v*) methanol for protein induction (Figure 1). After growth at 30 °C for three days, the plates were stained with Congo Red and further de-stained with NaCl until pale orange zones appeared around the colonies. Degradation halos bigger than the control (ct, *Pichia pastoris* transformed with empty pPICZαA vector) emerged around all the tested colonies, except for number 4. This indicated expression of a functional enzyme that can degrade CM-Cellulose. Colony PCR also confirmed positive transformants.

### 2.2. Af-EGL7 Expression and Purification

**Figure 2:**
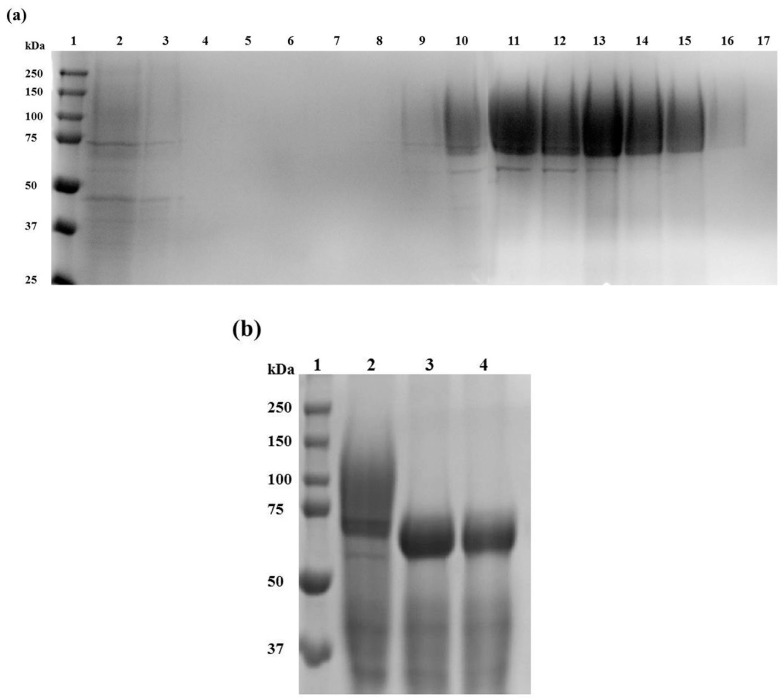
SDS-PAGE electrophoresis to evaluate (**a**) Af-EGL7 expression and purification. Lane 1: Molecular weight marker (Precision™ Protein Standards Dual Color; BioRad, Hercules, CA, USA). Lane 2: Flow-through. Lanes 3–7: Fractions obtained after elution with 0 mM imidazole. Lanes 8–17: Fractions obtained after elution with 5, 10, 20, 40, 40, 80, 80, 160, 250, and 500 mM imidazole. (**b**) Af-EGL7 deglycosylation. Lane 1: Molecular weight marker (Precision™ Protein Standards Dual Color; BioRad). Lane 2: Untreated purified Af-EGL7. The target protein is located with an estimated mass of 70 kDa. Lane 3 and 4: Af-EGL7 after Endo H treatment under non-denaturing and denaturing conditions, respectively. Both deglycosilation reactions produced a purified protein with approximately 60 kDa.

For this study, a positive *P. pastoris* X-33 transformant was selected, and Af-EGL7 was successfully expressed under control of the AOX-1 promoter after induction with 1.5% (*v*/*v*) methanol for six days. The culture supernatant was harvested by centrifugation and concentrated by tenfold before Af-EGL7 was purified by Ni^2+^-nitrilotriacetic (Ni-NTA) affinity chromatography. During protein elution, all fractions were collected and analyzed by SDS-PAGE (Figure 2a). Af-EGL7 eluted from 20 mM imidazole in greater amounts, but the fraction eluted with 80 mM imidazole had higher purity. All the fractions were combined, and the pure protein concentration estimated by the Greenberg method [18] was 450 µg protein·mL^−1^.

A previous study had indicated that the Af-EGL7 molecular weight is 48 kDa. However, the Af-EGL7 molecular weight estimated by SDS-PAGE herein was approximately 70 kDa. This difference suggested the presence of *N*-glycans in the Af-EGL7 structure, which agreed with the analysis performed by NetGlyc 1.0 Server (http://www.cbs.dtu.dk/services/NetNGlyc/) predicting four potential *N*-glycosylation sites in the Af-EGL7 structure, at the N96, N201, N205, and N379 residues [4].

Af-EGL7 treatment with Endoglycosidase H produced a protein with a molecular weight of approximately 60 kDa (Figure 2b). However, the molecular mass was still higher than the theoretical value even after deglycosylation. This difference could be due to protein hyperglycosylation, which is common in *P. pastoris* heterologous proteins, and to *O*-glycosylations, which usually occur in GH7 enzymes [19,20].

### 2.3. Biochemical Characterization

#### 2.3.1. Enzyme Activity

In a previous work, our group characterized recombinant Af-EGL7 expressed in *E. coli* and found that this enzyme displays optimal activity at 55 °C and pH 5.0 [4]. Here, recombinant Af-EGL7 expressed in *P. pastoris* also presented optimal activity under these same conditions.

In the present work, the Af-EGL7 V*_max_*, K*_M_*, and k*_cat_* kinetic parameters were determined at 55 °C by using CM-Cellulose, β-glucan, or Xyloglucan as substrates (Table 1). Af-EGL7 exhibited much higher activity in medium-viscosity barley β-glucan than in low-viscosity CM-Cellulose. This is an intrinsic feature of most GH7 endoglucanases and may be due to the high number of methoxy side chains substituting the CM-Cellulose, which can interfere in enzyme action. It also suggests that Af-EGL7 can hydrolyze both β-1,4- and β-1,3-glycosydic linkages [4,9].

Comparison of these kinetic parameters with those previously reported for the recombinant Af-EGL7 expressed in *E. coli* [4] revealed high Af-EGL7 catalytic efficiency after its expression in *P. pastoris*, which probably resulted from correct enzyme processing in the eukaryotic strain. The k*_cat_*/K*_M_* values in CM-Cellulose and β-glucan calculated for the recombinant Af-EGL7 expressed in *P. pastoris* were 995- and 1419-fold higher than the respective values obtained for the recombinant Af-EGL7 expressed in *E. coli*, respectively.

Furthermore, Af-EGL7 presented higher V*_max_* values than most endoglucanases described to date. Actually, the Cel7A V*_max_* is 5000 ± 186 μmol·min^−1^·mg^−1^ in the substrate barley β-glucan [9]. Hua and collaborators estimated a V*_max_* of 12.60 ± 0.64 μg·min^−1^·mL^−1^ for Ctendo7 in this same substrate [5]. The V*_max_* values reported for Egl7A and *Mt*Eg7A in CMC are 2257 ± 79 and 622.5 ± 86.4 μmol·min^−1^·mg^−1^, respectively, while the estimated value for an *A. terreus* GH12 endoglucanase is 16.15 μmol·min^−1^·mg^−1^ [11,21,22].

The effect of different ions and metals on recombinant Af-EGL7 expressed in *P. pastoris* was also tested herein. The same effect described by Bernardi et al. (2018) occurred [4]. However, considering the important role played by Cu^2+^ in lytic polyssacharide monooxygenase (LPMO) activity and the synergistic effect that LPMO and endoglucanases have on biomass hydrolysis, Af-EGL7 activity was also assessed at different Cu^2+^ concentrations (Table 2) [23].

Af-EGL7 activity increased at low Cu^2+^ concentrations (0.1–1.0 mM). Addition of 5, 10 and 15 mM Cu^2+^ partially reduced Af-EGL7 activity to 85%, 49%, and 21%, respectively. These results showed that the association of Af-EGL7 with LPMOs (copper-dependent enzymes) in an enzymatic cocktail is possible, which could enhance cellulose hydrolysis.

#### 2.3.2. Enzyme Stability

CAZymes (carbohydrate-active enzymes) glycosylation has been associated with higher enzyme thermal stability during natural and industrial processes, cellulose hydrolysis or binding, and protection from proteolysis [20,24]. In this sense, herein Af-EGL7 thermal stability was evaluated by pre-incubating the enzyme at different temperatures (55, 60, 70, 80 and 90 °C) for different time intervals.

**Figure 3:**
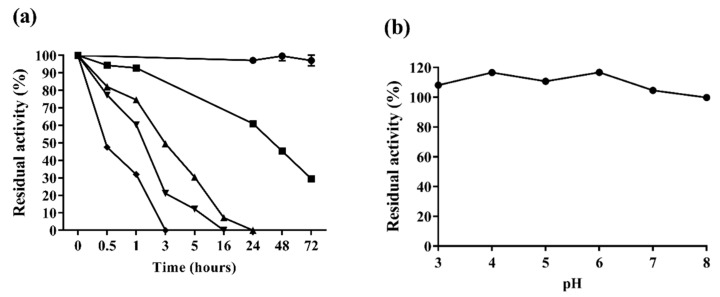
Plots of Af-EGL7 residual activity against incubation time at different temperatures and pH. (**a**) Af-EGL7 stability at ● 55 °C, ■ 60 °C, ▲ 70 °C, ▼ 80 °C, and ♦ 90 °C. (**b**) Af-EGL7 stability in the pH range 3.0–8.0. Error bars represent standard deviation (*n* = 3).

At 55 °C, the enzyme did not lose activity after incubation for 72 h. However, the enzyme started to lose activity at higher temperatures and retained 61% of its maximal activity after 24 h at 60 °C. After one hour at more extreme temperatures such as 70, 80 and 90 °C, the residual activities were 75%, 60% and 32%, respectively (Figure 3a).

The comparatively high temperature stability of the enzyme obtained here was surprising. When Af-EGL7 was expressed in *E. coli*, stability at these temperatures decreased rapidly, and residual activity was absent after 30 min at 60–90 °C [4]. Again, the better thermal stability observed in the present study was probably due to enzyme processing during its expression in *P. pastoris*, including post-translational modifications like glycosylation [15,16].

Cellulases from thermophilic microorganisms have commonly been described as having a high thermostability [25,26]. The endoglucanases *Mt*EG7a and Ctendo7 from the thermophilic fungi *M. thermophila* and *C. thermophilum* displayed about 40% and 60% residual activity after eight and one hour at 80 °C, respectively [5,11]. Egl7A and Cel7A from *T. emersonii* and *N. fisheri* retained more than 40% and 16% of their maximal activity after they were incubated at 70 °C for 1 h [9,27] (Table 3).

Our results revealed that Af-EGL7 was remarkably thermostable and withstood high temperatures even in the absence of a substrate. In fact, Af-EGL7 exhibited higher thermal stability than many previously reported thermostable glucanases. Given the numerous advantages of carrying out processes at temperatures above 50 °C, thermostability is one of the most desirable enzymatic properties in the industry [13,28,29]. Steam pre-treatment has been the most frequently employed treatment to make biomass suitable for enzymatic saccharification. Thermostable enzymes can be directly used after this heating step, saving time, expenses, and as well as providing better reaction yields [11,29,30,31].

Considering the broad range of biotechnological applications that β-glucanases have, great attention has also been directed to obtaining enzymes that are stable over a wide range of pH values. Alkaline-stable cellulases can be primarily applied as additives in washing powder and detergents. Cellulases that are stable under mild conditions (pH 5.0–6.0) can be used in brewing and biofuel production processes [4,11,32]. Acidophilic and acid-stable enzymes are favorable for application in the food and textile industries [16,32]. For this reason, here Af-EGL7 stability was evaluated at a pH ranging from 3.0 to 8.0. The recombinant enzyme was remarkably stable in both acidic and alkaline conditions (Figure 3b).

Analysis of the pH stability after incubation for 72 h did not show any Af-EGL7 activity loss. On the basis of these results, Af-EGL7 exhibited higher pH stability as compared to all the GH7 described so far, for which pH stability was only detected after shorter incubation intervals, as described in Table 3. Therefore, Af-EGL7 can be used in industrial processes over a broad pH range and for long incubation times.

#### 2.3.3. Af-EGL7 Tolerance to Product Inhibition

Cellobiose and glucose accumulation is one of the factors that limit biomass hydrolysis the most because it reduces enzyme catalytic efficiency and may increase process costs [34,35,36].

Previously, we described Af-EGL7 activity in xyloglucan [4]. The xyloglucan backbone resembles a cellulose chain: it consists of D-glucan (β1→4) substituted with xylose sidechains, which allows the products’ inhibitory effect on Af-EGL7 cellulase activity to be evaluated with precision [32]. In this sense, the effect of different glucose and cellobiose concentrations on Af-EGL activity was evaluated herein by using the chromogenic substrate Azo-Xyloglucan from Tamarind.

**Figure 4:**
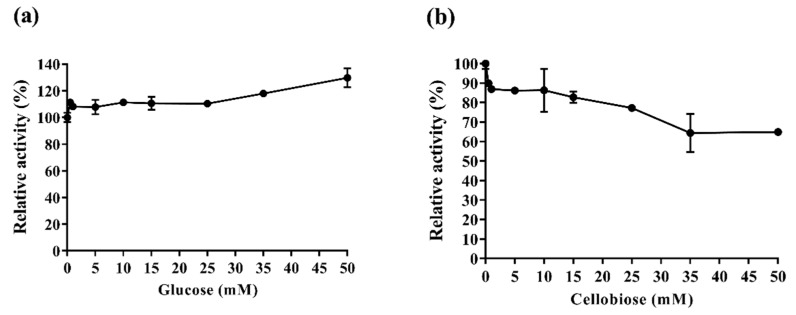
Glucose (**a**) and cellobiose (**b**) effect on Af-EGL7 activity. Error bars represent the standard deviation (*n* = 3).

Addition of glucose did not affect Af-EGL7 and even enhanced its activity at concentrations higher than 25 mM (Figure 4a). Moreover, 10 mM and 25 mM cellobiose inhibited Af-EGL7 activity by 14% and 23%, respectively. At the highest concentration of this effector (50 mM), residual activity corresponded to 65% (Figure 4b). Similar results were observed for the GH5 endoglucanase Egst: 30 mM glucose stimulated its activity by 1.6-fold, whereas 50 mM cellobiose inhibited its activity by about 33% [34].

These characteristics are highly important and desirable for the application of enzymes during biomass hydrolysis and make Af-EGL7 an enzyme of great commercial interest.

### 2.4. Enzyme Performance in Agroindustrial Residue Degradation

**Figure 5:**
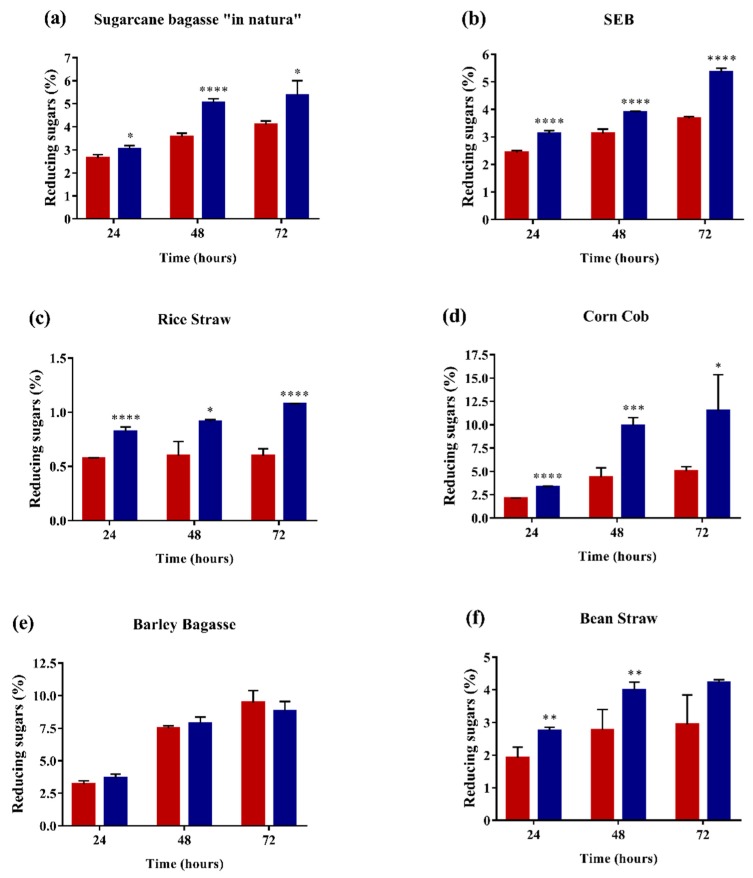
Effect of the cellulase cocktail Celluclast^®^ 1.5L (Red box) and supplemented by the addition of Af-EGL7 (Blue box), during hydrolysis of the agricultural residues (**a**) Sugarcane bagasse “in natura”, (**b**) SEB, (**c**) Rice straw, (**d**) Corn cob, (**e**) Barley bagasse and (**f**) Bean straw. The data represent means ± standard errors obtained from at least three experimental replicates.

To analyze the enzyme performance in biomass degradation, 1% of sugarcane bagasse “in natura”, sugarcane exploded bagasse (SEB), rice straw, corncob, barley bagasse or bean straw was added to 0.009 FPU of cellulase cocktail (Celluclast^®^ 1.5L) and 10 µg of thermostable Af-EGL7 and incubated at 55 °C and 1000 rpm for 24, 48, or 72 h. Interestingly, this enzyme showed a high degree of synergy with the cocktail in the deconstruction of all the tested substrates, except barley bagasse (Figure 5).

Af-EGL7 greatly enhanced the release of reducing sugars over the incubation time. Af-EGL7 synergy was higher in corncob and rice straw as substrates, which increased the amount of released reducing sugars by 128% and 80% after 72 h, respectively, as compared to cocktail alone.

Interesting facts were observed during SEB and sugarcane bagasse “in natura” hydrolysis. Surprisingly, when SEB hydrolysis was performed with the recombinant enzyme alone, released reducing sugars reached up to 1.3% ± 0.07 after 72 h Besides that, the cocktail hydrolyzed both SEB and sugarcane bagasse “in natura”, which had their conversion enhanced by Af-EGL7 at a similar rate (40–45%) after 72 and 48 h of reaction, respectively.

Finally, our data showed that Af-EGL7 was able to improve the hydrolysis of most of the evaluated agricultural residues, which have different compositions (Table S1, [37,38,39,40]), revealing a significant enzymatic potential for the industry in terms of efficiency and economy.

## 3. Materials and Methods

### 3.1. Strains, Culture Conditions and Vector

*Aspergillus fumigatus* Af293, kindly donated by Prof. Dr. Sérgio Akira Uyemura (University of São Paulo, Ribeirão Preto, Brazil), was grown in yeast extract-agar-glucose (YAG) medium (2.0% (*w*/*v*) dextrose, 2.0% (*w*/*v*) agar, 0.5% (*w*/*v*) yeast extract, and 0.1% (*v*/*v*) trace elements) at 37 °C for two days to obtain a fresh conidium suspension. The conidia were inoculated to a final concentration of 2 × 10^6^ per mL of YNB minimal medium (1× salts solution, 0.1% (*v*/*v*) trace elements, and 0.05% (*w*/*v*) yeast extract) containing 1% (*w*/*v*) fructose and incubated at 37 °C and under shaking at 200 rpm for 16 h. Next, the mycelia were harvested, washed, and transferred to YNB medium containing 1% (*w*/*v*) sugarcane exploded bagasse (SEB) at 37 °C and 200 rpm for 24 h. Then, the mycelia were harvested for RNA extraction.

The plasmid pPICZαA (Invitrogen, Carlsbad, CA, USA) was used for gene cloning, sequencing, and expression. *Escherichia coli* DH10β grown at 37 °C and 200 rpm in low salt Luria–Bertani medium supplemented with zeocin (50 µg·mL^−1^) was used to propagate the recombinant vector pPICZαA/*Af-egl7*. *Pichia pastoris* strain X-33 (Invitrogen, Carlsbad, CA, USA) cells harboring the recombinant expression vector pPICZαA/*Af-egl7* were used to produce heterologous protein. The employed growth conditions are described in the EasySelect™ *Pichia* Expression Kit manual (Invitrogen, Carlsbad, CA, USA).

The low-viscosity substrate CM-Cellulose was purchased from Sigma (Sigma–Aldrich, St. Louis, MO, USA). Medium-viscosity Barley β-glucan and Xyloglucan from tamarind seed were acquired from Megazyme (Megazyme International, Bray, Co. Wicklow, Ireland). The natural substrate Sugarcane Exploded Bagasse (SEB) was kindly provided by Prof. Dr. João Atílio Jorge (University of São Paulo). Sugarcane bagasse “in natura” was provided by Prof. Dr. Delia Rita Tapia Blácido (University of São Paulo, Ribeirão Preto, Brazil). Rice straw, bean straw, barley bagasse, and corncob were provided by Prof. Dr. Maria de Lourdes Teixeira de Moraes Polizeli (University of São Paulo, Ribeirão Preto, Brazil).

### 3.2. RNA Extraction, cDNA Synthesis, and Gene Amplification

*A. fumigatus* mycelia were collected after growth, as described above. After freezing in liquid nitrogen and grinding into a fine powder, total RNA was isolated by using the Direct-zol^TM^ RNA MiniPrep kit (Zymo Research, Irvine, CA, USA), according to the manufacturer’s instructions. cDNA was synthesized by using SuperScript^®^ II Reverse Transcriptase (Invitrogen, Carlsbad, CA, USA).

Specific primer sequences containing overlapping regions between the vector and the insert were employed (F: 5′-GAGAAAAGAGAGGCTGAAGCTGAATTCCAACAACCCGCCGCG-3′ and R: 5′-ATCCTCTTCTGAGATGAGTTTTTGTTCTAGCAGACACTGAGAGTA-3′; overlapping sites are underlined). The amplification reaction was performed with *Phusion High-Fidelity DNA Polymerase* (Thermo Fisher Scientific, Waltham, MS, USA) by using the follow thermocycling conditions: 98 °C for 30 s; 30 cycles of 98 °C for 10 s, 55 °C for 30 s, and 72 °C for 1 min; and 72 °C for 10 min. The PCR product was analyzed by electrophoresis and purified from 1% (*w*/*v*) agarose gel by using the QIAquick Gel Extraction kit (Qiagen, Hilden, Germany).

### 3.3. Cloning, Transformation of P. pastoris, and Screening of Recombinant Transformants

The ORF, without predicted signal peptide, was cloned into the vector pPICZαA (previously digested with the restriction enzymes *Eco*RI and *Xba*I) by the Circular Polymerase Extension Cloning (CPEC) method [17]. The CPEC reaction was performed with *Phusion High-Fidelity DNA Polymerase* (Thermo Fisher Scientific, Waltham, MS, USA); the thermocycling conditions used were as follows: 98 °C for 30 s; 35 cycles of 98 °C for 10 s, 55 °C for 30 s, and 72 °C for 2 min 30 s; and 72 °C for 10 min. The cloning product was transformed into *E. coli* DH10β, and the resistant transformants were selected by zeocin (50 µg·mL^−1^). The recombinant expression vector pPICZαA/*Af-egl7* was linearized with the restriction enzyme *Pme*I and transformed into *P. pastoris* X-33 competent cells by electroporation, according to the EasySelect™ *Pichia* Expression Kit manual (Invitrogen, Carlsbad, CA, USA).

Recombinant transformants with high-level endoglucanase expression were screened in yeast extract-peptone-dextrose (YPD) plates containing 1% (*w*/*v*) low-viscosity carboxymethylcellulose (CM-Cellulose), zeocin (100 µg·mL^−1^), and 1% (*v*/*v*) methanol for induction. After incubation at 30 °C for 3 days, the plates were stained with 0.1% (*w*/*v*) Congo red solution for 20 min and de-stained with 1 M NaCl until pale orange hydrolysis zones appeared against an orange background (approximately 20 min).

### 3.4. Recombinant Af-EGL7 Heterologous Expression in P. pastoris

A single colony of recombinant *P. pastoris* X-33 harboring the vector pPICZαA/*Af-egl7* was inoculated in buffered glycerol-complex medium (BMGY) (2% (*w*/*v*) peptone, 1.34% (*w*/*v*) yeast nitrogen base, 1% (*w*/*v*) yeast extract, 1% (*v*/*v*) glycerol, 4 × 10^−5^ % (*w*/*v*) biotin, 100 mM potassium phosphate buffer, pH 6.0) and grown at 30 °C and 240 rpm until the culture reached log phase growth (O.D._600nm_ = 2–6). Then, the cells were harvested by centrifugation at 3000× *g* for 5 min and resuspended in buffered methanol-complex medium (BMMY) (2% (*w*/*v*) peptone, 1.5% (*v*/*v*) methanol, 1.34% (*w*/*v*) yeast nitrogen base, 1% (*w*/*v*) yeast extract, 4 × 10^−5^ % (*w*/*v*) biotin, 100 mM potassium phosphate buffer, pH 6.0) to an O.D._600nm_ = 1. Af-EGL7 expression was induced at 30 °C and 240 rpm for 6 days (optimal time) under the control of the AOX1 promoter. Additional 1.5% (*v*/*v*) final concentration methanol was added to the medium every 24 h to maintain the expression levels. Once the *Af-egl7* gene was fused with the α-factor signal sequence, the recombinant protein was obtained extracellularly.

### 3.5. Recombinant Af-EGL7 Purification

After 6-day culture, the culture supernatant was collected by centrifugation at 3000× *g* for 5 min and concentrated to the maximum by using an Amicon Ultra-15 Centrifugal Filter—10 kDa cutoff (Millipore, Burlington, MS, USA). Next, the concentrate was resuspended in 20 mM sodium phosphate buffer containing 500 mM NaCl (pH 7.4) and loaded onto Ni Sepharose 6 Fast Flow resin (Ge Healthcare, Little Chalfont, United Kingdom) pre-equilibrated with the same buffer. After incubation at 4 °C for 1.5 h under stirring, a linear gradient from 0 to 500 mM imidazole in 20 mM sodium phosphate buffer containing 500 mM NaCl (pH 7.4) was applied to the column to elute the His_6_-tagged recombinant endoglucanase. All the fractions were collected, and protein was analyzed by 10% (*w*/*v*) SDS-PAGE, stained with Comassie Brilliant Blue R-250 (Sigma–Aldrich, St. Louis, MO, USA).

The fractions containing purified Af-EGL7 were mixed and submitted to buffer-exchange by using Amicon Ultra-15 Centrifugal Filter—10 kDa cutoff to remove excess imidazole prior to the subsequent enzymatic assays.

### 3.6. Endoglucanase Activity Assay

Af-EGL7 activity was determined by measuring reducing sugars from the reaction by the 3,5-dinitrosalicylic acid (DNS) method [41]. The enzymatic reactions were performed as described by Bernardi et al., 2018 [4]. Briefly, the reaction mixture consisting of 1% (*w*/*v*) CM-Cellulose in 50 mM sodium acetate buffer (pH 5.0) was incubated at 55 °C for 10 min. The enzyme action was stopped by adding an equal volume of the DNS reagent. The mixture was boiled for 5 min and cooled down, and the absorbance was measured at 540 nm. One unit of endoglucanase activity was defined as the amount of enzyme that released 1 µmol of reducing sugar from the substrate per minute. Each assay was carried out in triplicate. Protein concentration was determined by the Greenberg method [18].

The effect of different Cu^2+^ concentrations (from 0 to 15 mM) on the Af-EGL7 activity was also tested. The reactions were performed as described above.

### 3.7. Af-EGL7 Deglycosylation by Endo H

Purified Af-EGL7 deglycosylation was accomplished by Endoglycosidase H (Endo H, New England Biolabs, Ipswich, MA, USA) in both denaturing and non-denaturing conditions, according to the manufacturer’s instructions. In the first case, the recombinant protein was initially denatured at 100 °C for 10 min. Then, the deglycosylation reaction was performed at 37 °C for 24 h, as described by Meleiro et al. (2017) [34]. Proteins were further analyzed by SDS-PAGE.

### 3.8. Af-EGL7 Stability Assays

Thermostability was determined by pre-incubating 0.05 µg of purified Af-EGL7 in 50 mM sodium acetate buffer (pH 5.0) without substrate, at temperatures ranging from 55 to 90 °C for durations ranging from 30 min to 72 h. The residual activities were measured under standard conditions (pH 5.0, 55 °C, 10 min), as described in Section 3.6. The pH stability was estimated by measuring the residual enzymatic activity under standard conditions after incubation of the enzyme without substrate in Mcllvaine (citrate–phosphate) buffers pH 3.0–8.0 at 4 °C for up to 72 h [4].

### 3.9. Determination of Kinetic Parameters

The Af-EGL7 kinetic parameters (K_M_, V_max_, and k_cat_) were determined when CM-Cellulose (2.5–30 mg·mL^−1^), β-glucan (0.5–15 mg·mL^−1^), or xyloglucan (1.0–8.5 mg·mL^−1^) were used as substrates. The reactions were performed in 50 mM sodium acetate buffer (pH 5.0) as previously described. The parameters were calculated by linear regression, by using the Lineweaver–Burk graphical method.

### 3.10. Glucose and Cellobiose Effect on Af-EGL7 Activity

The glucose and cellobiose effect on Af-EGL7 activity was determined in the presence of increasing concentrations (0.5–50 mM) of both sugars by using the chromogenic substrate Azo-Xyloglucan from Tamarind (Megazyme International, Bray, Co. Wicklow, Ireland). The enzymatic assays were performed in 85 mM sodium acetate buffer (pH 4.5) at 40 °C for 10 min, according to the manufacturer’s instructions, with slight modifications. The reactions were stopped by adding 1.7 volumes of absolute ethanol. The supernatants were harvested by centrifugation at 1000× *g* for 10 min, and the absorbances were measured at 590 nm. Enzyme activity without glucose or cellobiose addition was considered 100%.

### 3.11. Lignocellulosic Biomass saccharification

Lignocellulose enzymatic hydrolysis was carried out as described previously by Bernardi et al. (2018) with some modifications [4]. The saccharification was accomplished in 50 mM sodium acetate buffer (pH 5.0) containing 1% (*w*/*v*) of one of the following biomasses: sugarcane bagasse “in natura”, SEB, rice straw, corncob, barley bagasse, or bean straw.

The reactions consisted of 0.009 FPU Celluclast^®^ 1.5L (Sigma–Aldrich, St. Louis, MO, USA) and 10 µg of Af-EGL7, added per 10 mg of each biomass. The reactions were conducted at 55 °C and 1000 rpm for up to 72 h in a final volume of 1 mL. DNS was added to stop the reactions and to measure the released reducing sugars. Control experiments were conducted in the same way, in the absence of Af-EGL7. The reported results represent the means ± SD calculated from at least three experimental replicates.

### 3.12. Statistical Analysis

Data are expressed as the mean of replicates ± SD. Significant differences between the treatment groups were analyzed by using Tukey’s test (significance, *p* < 0.05).

### 3.13. Reproducibility of the Results

All the data are the mean of at least three independent experiments and show consistent results.

## 4. Conclusions

At the industrial level, it is essential that enzymes maintain their activity at high temperatures and high pH values. This study has shown a GH7 endoglucanase with favorable properties for industrial applications, such as high thermal and pH stabilities. In addition, Af-EGL7 can be considered as a good hydrolytic enzyme that can improve biomass degradation by Celluclast^®^ 1.5L. Besides that, Af-EGL7 is a glucose-cellobiose-tolerant endoglucanase. All these properties make Af-EGL7 an important candidate for application in the improvement of commercial cocktails used in second-generation biofuel production as well as in other biorefinary processes.

## Figures and Tables

**Table 1:** Af-EGL7 kinetic parameters.

Substrate	K*_M_* (mg·mL^−1^)	V*_max_* (µmol·min^−1^·mg^−1^)	k*_cat_* (s^−1^)	k*_cat_*/K*_M_* (mL·mg^−1^·s^−1^)
CM-Cellulose	24.5 ± 0.6	6193 ± 140	5037 ± 114	205.9 ± 0.8
β-glucan	5.6 ± 0.3	13722 ± 519	11161 ± 422	1992 ± 37
Xyloglucan	8.6 ± 0.3	9082 ± 293	7386 ± 238	859.9 ± 0.2

**Table 2:** Effect of different Cu^2+^ concentrations on Af-EGL7 activity.

Cu^2+^ Concentration (mM)	Af-EGL7 Relative Activity (%)
**0.0**	100.0 ± 1.7
**0.1**	107.2 ± 1.4
**0.5**	115.5 ± 0.5
**1.0**	117.4 ± 0.7
**5.0**	85.9 ± 1.5
**10.0**	49.8 ± 1.5
**15.0**	21.7 ± 0.3

**Table 3:** Comparison among the catalytic and stability properties of GH7 endoglucanases from different organisms.

Source Organism	Expression System	Substrate	V*_max_* (U·mg^−1^)	K*_M_* (mg·mL^−1^)	k*cat* (s^−1^)	k*cat*/K*_M_* (mL·mg^−1^·s^−1^)	Thermal Stability	pH Stability
*Aspergillus fumigatus* (this work)	*Pichia pastoris*	β-Glucan	13722 ± 519	5.6 ± 0.3	11161 ± 422	1992 ± 37	100% after 72 h at 55 °C; 30% after 1 h at 90 °C	No loss after 72 h in the pH range 3.0–8.0
		Xyloglucan	9082 ± 293	8.6 ± 0.3	7386 ± 238	859.9 ± 0.2		
		CMC-Na	6193 ± 140	24.5 ± 0.6	5037 ± 114	205.9 ± 0.8		
*Aspergillus fumigatus* [4]	*Escherichia coli*	β-Glucan	191.9 ± 0.0007	113.9 ± 0.005	159.9	1.4	85% after 48 h at 50 °C; 40% after 1 h at 55 °C	Start to decrease after 24 h at pH 6.0
		CMC-Na	51.9 ± 0.007	209.7 ± 0.1	43.3	0.2		
*Neosartorya fischeri* [9]	*Pichia pastoris*	β-Glucan	5000 ± 186	4.5 ± 0.2	-	-	16.1% after 1h at 70 °C	No loss after 1 h in the pH range 3.0–8.0
*Talaromyces emersonii* [27]	*Pichia pastoris*	β-Glucan	17951 ± 69	4.0 ± 1.5	-	3156 ± 24	More than 50% after 2 min at 80 °C	More than 70% and 50% after 1 h in the pH range 1.0–11.0 and at pH 12.0, respectively
		CMC-Na	2257 ± 79	20.8 ± 2.1	-	78.9 ± 3.8		
*Myceliophthora thermophile* [11]	*Pichia pastoris*	CMC-Na	622.5 ± 86.4	24.0 ± 0.5	-	0.313667	t_1/2_ = 9.96 h (70 °C); t_1/2_ = 6.5 h (80 °C)	No loss after 24 h in the pH range 3.0–11.0
*Trichoderma harzianum* [12]	*Aspergillus niger*	Xyloglucan	0.22 ± 0.095 (μM·s^−1^)	1.98 ± 0.47	0.45	-	100% after two months of incubation at 55 °C and pH 5.0	-
*Chaetomium thermophilum* [5]	*Pichia pastoris*	CMC-Na	59.6 ± 8.2	79.2 ± 5.8	2.11 × 10^−3^	0.02673	61.3% after 60 min at 80 °C; Almost all activity lost after 100 min at 90 °C	-
		β-Glucan	12.6 ± 0.6 (µg·min^−1^·mL^−1^)	9.8 ± 0.6	0.7 × 10^−3^	0.07849		
*Bispora sp.* MEY-1 [33]	*Pichia pastoris*	β-glucan	6737	9.16	-	-	100% after 1 h at 60 °C; More than 30% after 1 h at 70 °C	More than 85% after 1 h in the pH range 1.0–8.0
		CMC-Na	3460	287	-	-		

## Supplementary Materials

Supplementary materials can be found at https://www.mdpi.com/1422-0067/20/9/2261/s1.
