# Supplementary material for: A Thermostable Aspergillus fumigatus GH7 Endoglucanase Over-Expressed in Pichia pastoris Stimulates Lignocellulosic Biomass Hydrolysis
Source: Int J Mol Sci. 2019 May 7;20(9):2261. doi: 10.3390/ijms20092261 (PMC6540056; doi:10.3390/ijms20092261)
Supplement: Supplementary file 1 [file ijms-20-02261-s001.pdf]

Table S1. Lignocellulosic composition of biomasses

Biomass	Cellulose (%)	Hemicellulose (%)	Lignin (%)
Sugarcane bagasse “in natura” [37]	42.0	25.0	20.0
SEB [7]	47.5	9.0	34.3
Rice straw [38]	32.1	24.0	18.0
Corn cob [38]	45.0	35.0	15.0
Barley bagasse [39]	12.3	23.4	26.1
Bean straw [40]	43.7	28.0	8.4
